# Ultra-Wideband (UWB) Systems in Biomedical Sensing

**DOI:** 10.3390/s22124403

**Published:** 2022-06-10

**Authors:** Gianluigi Tiberi, Mohammad Ghavami

**Affiliations:** 1 School of Engineering, London South Bank University, London SE1 0AA, UK; tiberig@lsbu.ac.uk; 2 UBT—Umbria Bioengineering Technologies, 06081 Perugia, Italy

The extremely low power transmission levels of ultra-wideband (UWB) technology, alongside its advantageously large bandwidth, make it a prime candidate for being used in numerous healthcare scenarios, which require short-range high-data-rate communications and safe radar-based applications. UWB remote sensing systems are becoming extremely popular, especially for permitting non-invasive diagnosis and short- and long-term monitoring/surveillance of health conditions. Moreover, several unique properties of UWB, including its non-ionizing signals, low cost, and ability to penetrate through media (air, skin, bones, and tissues), have transformed it into an ideal candidate to be used as a novel medical imaging technology.

This Special Issue delivers original research on different aspects of UWB systems in biomedical sensing, including: UWB biosensing and biosignal analysis and processing for detection of the heart rate, respiratory movements, and human gait analysis; artificial intelligence (AI)-augmented data processing; UWB antennas and system electronics; UWB imaging for lesion detection.

Several UWB systems for biosensing and biosignal analysis, all performed in an unobtrusive way, are proposed for monitoring a person’s presence and/or collecting his/her health-related parameters simultaneously in a home environment [1,2,3]. Using UWB impulse-radar as a sensing device, the authors of [1] showed that it is possible to recognize a person’s presence and monitor and collect his/her health-related parameters (such as breathing and coughing rates) simultaneously in a home environment; in addition, they implemented a machine learning technique (k-nearest neighbor) to automatically classify a static posture using UWB radar data. Dedicated machine learning approaches were also employed in [2] to differentiate between different human emotions (such as happiness, disgust, and fear) using UWB impulse-radar data (without placing any on-body sensors), reaching an accuracy of 76%. Specifically, the authors included a total of 35 subjects in their study, inducing emotions (with dedicated videos) and collecting UWB impulse-radar data. A first attempt to use UWB for measuring the arterial blood pressure was presented in [3].

Novel and dedicated UWB sensor and system electronics for a variety of applications were investigated, tested, and optimized in [4,5]. In [4], the authors presented the validation of a new commercial contactless and continuous respiratory rate monitoring device; validation was carried out by direct comparison to manually scored reference data in a total of 50 subjects. It was shown that the accuracy rate reached 90%. A novel, compact, and low-cost UWB sensor for vital sign monitoring in pre-hospital settings was proposed in [5]. Specifically, the authors demonstrated the functionality of the sensor for respiration detection and heartbeat monitoring. Interestingly, with seventeen tests performed for respiration rate detection, sixteen of them were successfully detected. The idea is that this approach could be employed in ambulance settings, after having addressed the issues related to background vibration.

UWB medical imaging is a promising technique securing many benefits in providing low-risk imaging of the internal organs and tissues of the human body, exploiting the contrast in dielectric properties. Several UWB breast imaging devices have been constructed; a few of them have been clinically validated. Recently, UWB imaging has also been applied to brain stroke classification, bone imaging, and lung cancer detection. To further push performances, dedicated UWB antennas and switchable filters were proposed in [6,7]. Specifically, in [6], the authors proposed a highly efficient, low-cost, microstrip monopole antenna (which consists of stepped meander lines), achieving simulated and measured frequency bands of 2.7–22.5 GHz and 2.8–22.7 GHz, respectively. UWB antennas may also have a dedicated switchable filter. In this context, in [7], the authors investigated the time-domain performance of a filter antenna, showing that the switchable antenna has a good time-domain resolution and assessing its potential for breast imaging.

UWB imaging may be affected by artefacts, i.e., transmitter images and the reflections of the external layers, which might mask the inclusions, reducing the detection capability. In [8], the authors presented an investigation of different artefact removal methods for UWB imaging through measurements using a dedicated device; from their results, they concluded that the local average subtraction and the rotation subtraction are the best approaches to remove artefacts in terms of the signal-to-clutter ratio.

In conclusion, the papers included in this Special Issue confirm that UWB radar is a safe and powerful tool for non-invasive and non-intrusive measurements based on electromagnetic fields. UWB provides a good spatial resolution to distinguish different body postures, presence, coughing, breathing, and other activities. Thus, UWB applications in healthcare scenarios are growing, thanks to the continuous advances in hardware technology/sensors and in AI-based signal processing.
